# Assessment of Raftlin and blood count parameters in otosclerosis

**DOI:** 10.3389/fneur.2025.1660767

**Published:** 2025-09-03

**Authors:** Duygu Erdem, Sultan Şevik Eliçora, Berrak Güven, Deniz Baklaci

**Affiliations:** ^1^Department of Otorhinolaryngology, Faculty of Medicine, Zonguldak Bülent Ecevit University, Zonguldak, Türkiye; ^2^Department of Otorhinolaryngology, Kocaeli City Hospital, Kocaeli, Türkiye; ^3^Department of Medical Biochemistry, Faculty of Medicine, Zonguldak Bülent Ecevit University, Zonguldak, Türkiye

**Keywords:** otosclerosis, blood cell count, inflammation mediators, Raftlin, hearing-disorders

## Abstract

**Objective:**

Although otosclerosis is a common disease, its etiology has not yet been clearly elucidated. One of the factors recently implicated in its pathogenesis is chronic inflammation. Blood count parameters are known as classical inflammatory markers in several diseases. Raftlin is also a novel biomarker of inflammation. The present study aimed to investigate Raftlin levels and blood count parameters in otosclerosis.

**Methods:**

The study included 50 otosclerosis patients and 50 healthy volunteers. Serum Raftlin levels and white blood cell, neutrophil, lymphocyte and platelet counts, neutrophil to lymphocyte ratio (NLR), platelet to lymphocyte ratio (PLR), and mean platelet volume (MPV) were measured in otosclerosis patients and compared with the controls.

**Results:**

White blood cell and neutrophil counts and NLR were statistically higher in otosclerosis patients than in the control group (*p* < 0.001). There was no statistically significant difference between the control and patient groups in lymphocyte and platelet counts, and MPV and PLR values. The Raftlin level was also statistically significantly higher in otosclerosis patients than in the control group (*p* = 0.001).

**Conclusion:**

The Raftlin level and NLR value were significantly higher in otosclerotic patients than in healthy controls, supporting the presence of an inflammatory etiology for otosclerosis. Our study is the first in the literature to investigate the relationship between otosclerosis and Raftlin with known inflammatory markers.

## Highlights

- Otosclerosis is the primary disease of the human otic capsule and the stapes footplate.
- The role of active inflammation in the etiopathogenesis of the disease has been investigated.
- Raftlin, a main lipid raft protein on B cells, plays an important role in initiating a variety of inflammation responses.
- Raftlin levels and NLR values were found to be statistically significantly higher in the serum samples of otosclerosis patients when compared with normal population.

## Introduction

Otosclerosis is a disease in the human temporal bone with bone remodeling and new bone formation (1, 2). Depending on the affected area and histological types, it may cause conductive hearing loss and balance problems. Although the disease is common, the etiopathogenesis has not yet been completely elucidated (1–3). Genetic susceptibility, some disorders in bone metabolism, viral infections, autoimmune disorders, and various environmental or hormonal factors are implicated in the pathogenesis (1–5). There are also studies investigating the role of active inflammation in bone remodeling in the etiopathogenesis of the disease (6, 7).

Although the etiology of otosclerosis has not yet been completely elucidated yet, it has been shown by several studies that chronic inflammation might have a potential effect in the pathophysiology (8–10). Quesnel et al. (11) suggested that the primer pathology in otosclerosis was abnormal bony remodeling, including inflammation-induced bone resolution, new bone formation, and vascular proliferation in the otic capsule (11). Karosi and Sziklai (7) reported that there had been many reports confirming the association between otosclerosis and inflammation (7). Bone destruction and reorganization in otosclerosis are probably triggered by several inflammatory events. In immunohistochemical examinations, the determination of T lymphocytes, complementary fragments, and microglobulin in otosclerotic foci is considered to be evidence of an inflammatory mechanism in otosclerosis (8). Based on these data, we decided to investigate the role of inflammation in otosclerosis through various markers in the present study.

Complete blood count (CBC) and several related parameters are known to be classical markers of inflammation in many cardiovascular, inflammatory and oncological diseases (12, 13). The neutrophil to lymphocyte ratio (NLR), platelet to lymphocyte ratio (PLR), and mean platelet volume (MPV) are described as indicators of inflammation. They are routinely calculated as part of peripheral blood count analysis without any additional cost (8, 12, 14, 15). NLR and PLR have been previously examined in several otolaryngological diseases, such as sudden hearing loss, facial paralysis, chronic otitis media, serous otitis media, and salivary gland pathologies and accepted as a marker of inflammation (8, 16, 17). MPV is one of the parameters routinely reported in CBC analysis and shows increased and reduced levels in chronic inflammation (8, 18). MPV provides information about the platelet sizes and reflects a high platelet activity. Inflammatory and thrombotic cytokines in platelets increase as MPV increases. It has been previously emphasized that activated or large platelets are depleted in high-grade inflammatory conditions or defective thrombopoiesis due to inflammation, leading to decreased MPV. On the other hand, Raftlin is the major lipid raft protein on B cells and a novel defined marker (19). It acts as a signal transducer of the B cell receptor (BCR) and the T cell receptor (TCR) (19–21). Raftlin also has a meritorious role in initiating a variety of autoimmune and vascular inflammation responses (22, 23).

The present study aimed to investigate Raftlin levels and CBC parameters commonly used as an inflammatory marker in current practice in patients with otosclerosis. When we search the literature, we found no study investigating Raftlin levels together with already known inflammatory markers in otosclerosis.

## Methods

Ethics committee approval was received before the study (08/21/2019, decision number: 2019–12), all individuals signed an informed consent. The study group comprised 50 patients with otosclerosis who were operated in our clinic between January 2013 and January 2020. Patients were not included if they had a history of ear intervention or middle ear disease, any hematologic or coronary disease, chronic renal failure, inflammatory, autoimmune and infectious disease, or a history of cancer, or if they were receiving corticosteroid therapy. The control group consisted of 50 healthy individuals. Similar criteria administered to the patient group were also carried out for the control group. The diagnosis of otosclerosis was made by anamnesis, ear examination, and audiometry. In all 50 patients, the preliminary diagnosis was confirmed by the fixation of the footplate during the operation.

Blood samples were collected from all participants while they were prepared for the operation within the week before surgery and after a 12-h fasting period. The preoperative white blood cell (WBC), neutrophil, lymphocyte, and platelet counts, NLR, PLR, and MPV were simultaneously analyzed through a CBC test in the Biochemistry Department. For calculating NLR, the neutrophil count was divided to the lymphocyte count; and for PLR, the platelet count was divided to the lymphocyte count. The serum specimens were kept at −80 degrees until analysis after centrifugation of the blood samples. Then, Raftlin levels were measured based on Raftlin (Human-RFTN2), with commercial kits according to the manufacturer’s recommendations (Sun Red, Human RFTN-2 ELISA Kit, Shanghai, China).

Statistical evaluations were performed with PASW package program, version 19.0. Descriptive statistics were shown as mean ± standard deviation. *p* values of less than 0.05 were considered statistically significant for all tests. Whether the data showed normal distribution was determined with the Kolmogorov–Smirnov test. Differences in continuous variables between the otosclerosis and control groups were analyzed using the Mann–Whitney U test. The Chi-squared test was used for the comparison of categorical data, such as gender. Receiver operating characteristic (ROC) curve analysis was performed to evaluate the diagnostic performance of Raftlin and NLR. The area under the curve (AUC) was calculated to distinguish otosclerosis patients from healthy controls, and optimal cut-off values were determined.

## Results

The otosclerosis group included 19 male and 31 female patients, and the control group 22 male and 28 female patients. No statistically significant difference was observed between otosclerotic and the patient groups in terms of gender and age (*p* = 0.383). The demographic data of the otosclerosis and control groups are demonstrated in Table 1.

**Table 1:** The demographic values of the patient and control groups.

Gender	Otosclerosis group (*n* = 50)	Control group (*n* = 50)
Male	19 (38%)	22 (44%)
Female	31 (62%)	28 (56%)
Age (mean ± SD)	40.84 ± 11.43	42.92 ± 12.28

SD, Standard deviation.

When the two groups were compared for CBC parameters, statistically significantly higher results were found in the patient group for the WBC count, neutrophil count, and NLR (*p* < 0.001 for all). No statistically significant difference was detected in the lymphocyte and platelet counts, and MPV and PLR values between the otosclerosis and the control groups (*p* > 0.05). When the two groups were compared in terms of Raftlin levels, they were statistically significantly higher in the otosclerosis group (*p* = 0.001). The CBC parameters and Raftlin levels of the patient and control groups are presented in Table 2.

**Table 2:** Raftlin and complete blood count parameters of the patient and control groups.

Mean ± SD	Otosclerosis group (*n* = 50)	Control group (*n* = 50)	*p* value
Raftlin (ng/mL)	12.006 ± 11.5851314	4.9358 ± 2.6101529	0.001*
WBC (10^3^/IU)	8.84 ± 2.3754699	6.898 ± 1.3638915	<0.001*
Neutrophil count (10^3^/IU)	5.78 ± 1.9868958	3.562 ± 0.8007369	<0.001*
Lymphocyte count (10^3^/IU)	2.266 ± 0.7347386	2.312000 ± 0.5359104	0.112
Platelet count (10^3^/IU)	262.44 ± 68.8841559	247.28 ± 49.6156822	0.092
NLR	2.874009 ± 1.5951506	1.585217 ± 0.3636798	<0.001*
PLR	127.06 ± 61.2550406	111.76 ± 33.4929539	0.149
MPV (fl)	9.076 ± 1.1555651	8.780000 ± 0.9171829	0.208

*Statistically significant, *p* < 0.05, SD, Standard deviation; WBC, White blood cell count; NLR, Neutrophil to lymphocyte ratio; PLR, Platelet to lymphocyte ratio; MPV, Mean platelet volume.

**Figure 1:**
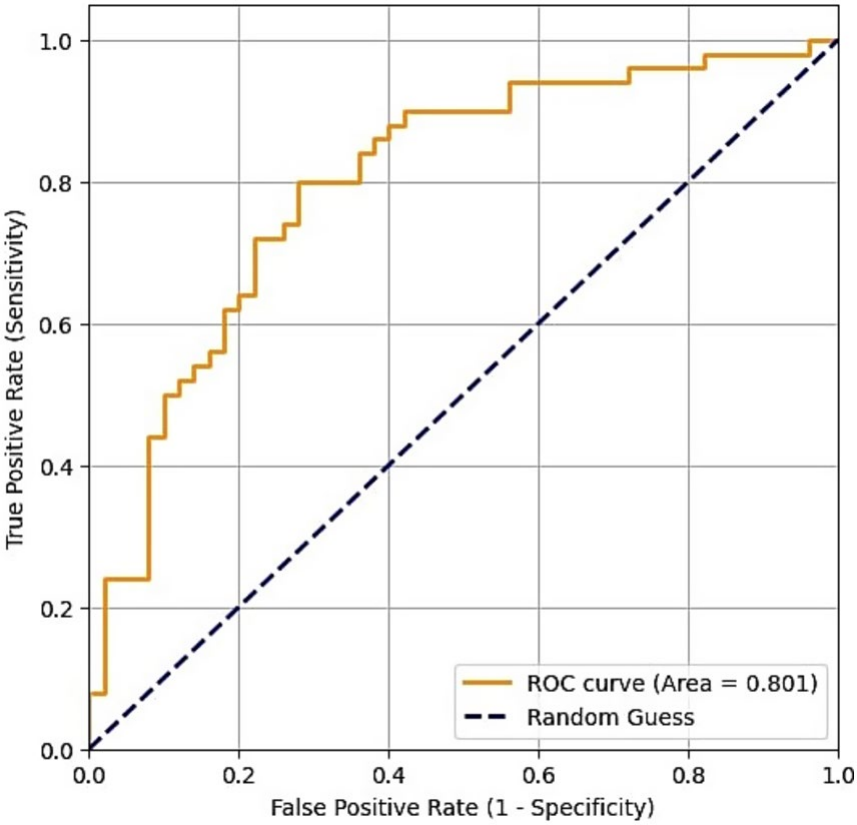
Receiver operating characteristic curve of Raftlin.

**Figure 2:**
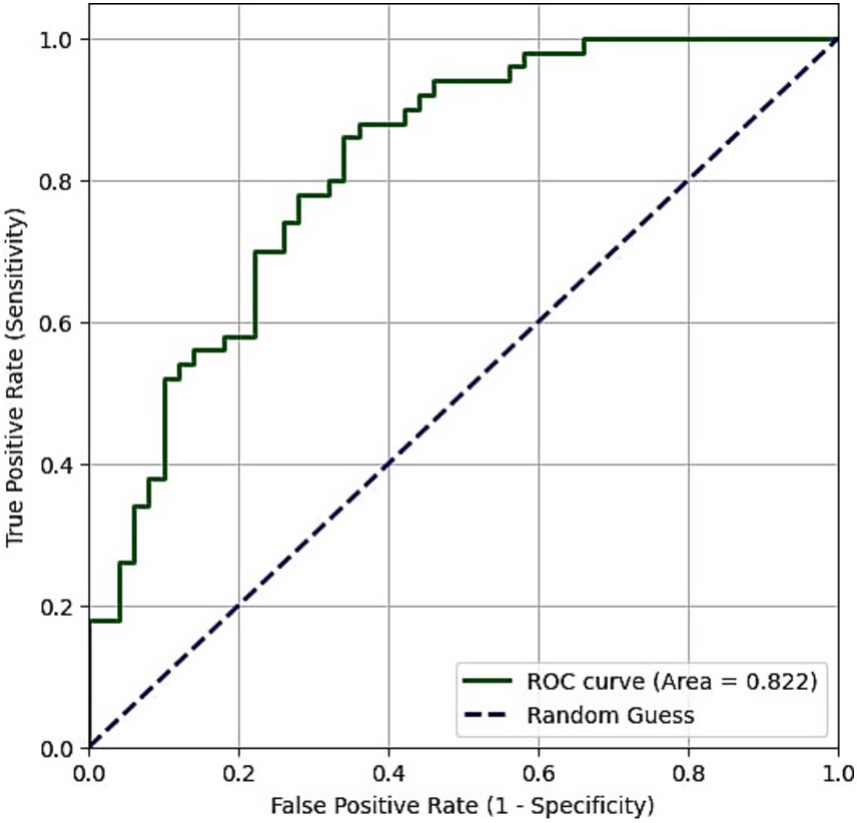
Receiver operating characteristic curve of NLR.

Receiver operating characteristic (ROC) curves were plotted for diagnostic measurements and cut-off values of both Raftlin and NLR. The area under the curve (AUC) for Raftlin was 0.694 (*p* = 0.001), and the cut-off value for otosclerotic patients was 4.55 ng/mL (Table 3 and Figure 1). For NLR, AUC was 0.846 (*p* < 0.001), and the cut-off value was 1.748876 (Table 3 and Figure 2).

**Table 3:** ROC analysis of Raftlin and NLR.

Parameter	AUC	95% CI	Cut-off	*p*
Raftlin	0.694	0.519–0.76	4.55 ng/mL	0.001*
NLR	0.846	0.745–0.906	1.748876	<0.001*

NLR, Neutrophil to lymphocyte ratio; AUC, Area under the curve; ROC, Receiver operating characteristic; 95% CI: 95% confidence of interval *: Statistically significant; *p* < 0.05.

## Discussion

The present study aimed to investigate serum Raftlin levels and CBC parameters in patients with otosclerosis and to compare with healthy volunteers. The outcomes showed that the Raftlin levels and NLR were statistically significantly higher in otosclerotic individuals than in the healthy population. The diagnostic value of these two markers for otosclerosis was also significant according to the ROC analysis. Our study is the first in the literature to analyze the relation between otosclerosis and Raftlin together with already known inflammatory markers.

Otosclerosis is a disease that progresses with a bone remodeling in human temporal bone and leads to conductive type hearing loss due to the of fixation in the stapedial footplate. It is a widespread disease seen twice as often in women as men. In our study, the demographic distribution of patients was consistent with the literature.

In a study by Ulu et al. (24) it is reported that in sudden sensorineural hearing loss patients NLR values were higher than in controls. In our present study, we similarly detected that NLR was statistically significantly higher in otosclerotic group than in the healthy population (*p* < 0.001). This finding supports the idea of the role of chronic inflammation in the etiology of otosclerosis. However, in a study conducted by Arli et al. (8), any statistically significant difference was not detected for the WBC, NLR, PLR, and platelet values between the otosclerosis and control groups, which can be attributed to the higher number of patients and controls in our sample.

In previous studies, platelet count and PLR values were found high in coronary artery diseases, chronic renal failure, and several malignancies (11). Unlike these studies, we found no significant difference between the otosclerotic patients and the control groups in among the platelet count and PLR values.

Several studies have shown a possible link between MPV and systemic or local inflammatory diseases (18). Yazici et al. (25) determined that MPV were statistically higher in rheumatoid arthritis patients than in the control group. They also reported that MPV values were directly linked with inflammatory markers and the prognosis of the disease (25). Somuk et al. (26) observed no statistical difference in MPV between the chronic serous otitis media patients and the control group (26). We also found no significant difference in MPV values among the patient group and healthy volunteers. However, Arli et al. (8) indicated that MPVs were lower in otosclerotic individuals compared to the healthy population (8). They reported that a decrease in MPV levels was associated with vascular abnormalities, inflammatory bowel diseases, chronic anemia or renal failure, appendicitis, pancreatitis, sepsis, and viral infections (RSV, EBV, HIV, rubella, etc.). Furthermore, some authors suggest that some viruses, for example rubella, measles, and RSV have a role in the pathogenesis of otosclerosis (8). In terms of MPV values, our results do not support viral etiology in contrast to the study of Arli et al. (8).

Although the specificity of Raftlin as a biomarker for otosclerosis may be limited due to its elevation in various inflammatory and systemic diseases, recent studies have demonstrated its relevance in several otolaryngological and non-otolaryngological conditions. It is a raft protein first introduced in Raji B cells, involved in the signal transmission of BCR. It also plays a role in autoimmune responses and the activation of TCR (22). In a previous study by Lee et al. (20), Raftlin was determined as a diagnostic marker for sepsis and septic shock (20). In another study, it was stated that Raftlin had a significant role in the wound healing process (23). According to Saeki et al. (19), Raftlin has a direct effect on lipid raft assembly and can change the amounts of rafts through its enzymatic activity (19). Lin et al. (27) reported that Raftlin expression in patients with chronic rhinosinusitis with nasal polyps was associated with increased levels of inflammatory cytokines such as IL-17 and TNF-α, suggesting a role in epithelial remodeling and chronic inflammation in the upper airway. Similarly, elevated Raftlin levels have been observed in neuropsychiatric disorders, including major depressive disorder (28) and schizophrenia (29), as well as in spinal pathologies like Modic changes (30). Furthermore, Raftlin has been shown to correlate with disease severity in sepsis, highlighting its potential as a general marker of inflammation (31). These findings support the notion that while Raftlin may not be specific to otosclerosis, its elevation in our patient group reinforces the hypothesis of an inflammatory component in the pathogenesis of the disease.

Intact lipid rafts and Raftlin are necessary for numerous activities, such as BCR activation and calcium mobilization. Saeki et al. (32) also reported that TCR signaling was positively affected by Raftlin, and over-expression of Raftlin could cause predisposition to the development of many T cell-dependent autoimmune diseases. In light of this information, we thought that Raftlin could play a meritorious role in the diagnosis of otosclerosis and decided to investigate it in patients with this disease. As expected, we found Raftlin levels to be statistically significantly higher in the otosclerotic group when compared with the healthy individuals (*p* = 0.001).

When we searched the literature, we found only one study that investigated CBC parameters in otosclerosis patients, and there was no research evaluating the levels of Raftlin. Since Raftlin is a novel biomarker, on which there are only a limited number of studies, we found it appropriate to screen other proven inflammatory biomarkers together with Raftlin when planning our study. We found statistically significantly higher results for both Raftlin and NLR in patients with otosclerosis than in the healthy population. Then, we performed a ROC analysis to investigate the diagnostic value of these parameters in the disease. We found the cut-off values as 4.55 ng/mL for Raftlin and 1.748876 for NLR.

Despite all developments, otosclerosis remains a disease full of unknowns. Even today, the diagnosis can only be made surgically or histopathologically, and medical treatment cannot be performed since the cause of sensory-neural hearing loss in advanced otosclerosis has not yet been completely resolved. If otosclerosis is proven to be an autoimmune or inflammatory disease, the use of new medical treatment options, including anti-inflammatory or immunosuppressant drugs can also be discussed (32). We consider that our study contributes to a better understanding of the disease by showing that inflammation has somewhat a meritorious role in the etiopathogenesis.

The limitations of our study include the relatively low number of participants in both control and patient groups, and the lack of an evaluation of the effect of NLR or Raftlin on pre-treatment hearing levels or treatment success in patients with otosclerosis. Obtaining these data can be the subject of further studies. While both NLR and Raftlin levels were significantly elevated in the otosclerosis group, the ROC analysis revealed a notable difference in their diagnostic utility. With an AUC of 0.846, NLR demonstrated good performance as a potential biomarker for otosclerosis, significantly outperforming Raftlin (AUC = 0.694). This suggests that the systemic inflammatory balance, as captured by the ratio of neutrophils to lymphocytes, may be a more robust indicator of the inflammatory processes in otosclerosis. Although the elevation in Raftlin is a novel and statistically significant finding, its modest AUC value indicates limited clinical utility as a standalone diagnostic test at this stage. However, this result is still valuable as it is the first to implicate the Raftlin-associated pathways in otosclerosis, potentially involving B-cell and T-cell mediated responses. This opens a new avenue for future research to explore the specific role of these autoimmune pathways in the disease’s pathogenesis, rather than presenting Raftlin as a definitive marker.

Otosclerosis is a widespread disease with a yet-to-be elucidated etiopathogenesis. In present study, Raftlin and NLR were statistically significantly higher in the serum specimens of the patients with otosclerosis compared with the healthy population. The results of the present study confirm that inflammation plays a meritorious role in the etiopathogenesis of otosclerosis. However, further controlled studies containing more patients are necessary to provide a better understanding of how inflammation affects the disease.

## Data Availability

The original contributions presented in the study are included in the article/supplementary material, further inquiries can be directed to the corresponding author.

## References

[1] Uppal S Bajaj Y Rustom I Coatesworth AP. Otosclerosis 1: the aetiopathogenesis of otosclerosis. Int J Clin Pract. (2009) 63:1526–30. doi: 10.1111/j.1742-1241.2009.02045.x, PMID: 19769709

[2] Yıldırım YS Apuhan T Düzenli S Arslan AO. Otosclerosis and vitamin D receptor gene polymorphism. Am J Otolaryngol. (2013) 34:454–7. doi: 10.1016/j.amjoto.2013.03.016, PMID: 23639864

[3] Quaranta N Piccininni K Romanello M Lucidi D Sergi B. The impact of intra-operative factors in otosclerosis outcomes: retrospective study in a tertiary center. Acta Otorhinolaryngol Ital. (2019) 39:197–204. doi: 10.14639/0392-100X-2004 31131839 PMC6536026

[4] Rudic M Keogh I Wagner R Wilkinson E Kiros N Ferrary E. The pathophysiology of otosclerosis: review of current research. Hear Res. (2015) 330:51–6. doi: 10.1016/j.heares.2015.07.014, PMID: 26276418

[5] Schrauwen I Van Camp G. The etiology of otosclerosis: a combination of genes and environment. Laryngoscope. (2010) 120:1195–202. doi: 10.1002/lary.20934, PMID: 20513039

[6] Liktor B Szekanecz Z Batta TJ Sziklai A Karosi T. Perspectives of pharmacological treatment in otosclerosis. Eur Arch Otorrinolaringol. (2013) 270:793–804. doi: 10.1007/s00405-012-2126-0 22843095

[7] Karosi T Sziklai I. Etiopathogenesis of otosclerosis. Eur Arch Otorrinolaringol. (2010) 267:1337–49. doi: 10.1007/s00405-010-1292-1, PMID: 20532905

[8] Arli C Gulmez I Saraç ET Okuyucu Ş. Assessment of inflammatory markers in otosclerosis patients. Braz J Otorhinolaryngol. (2019) 86:456–60. doi: 10.1016/j.bjorl.2018.12.014 30926454 PMC9422544

[9] Michaels L Soucek S. Origin and growth of otosclerosis. Acta Otolaryngol. (2011) 131:460–8. doi: 10.3109/00016489.2010.532156, PMID: 21142744

[10] Babcock TA Liu XZ. Otosclerosis, from genetics to molecular biology. Otolaryngol Clin N Am. (2018) 51:305–18. doi: 10.1016/j.otc.2017.11.002, PMID: 29502723

[11] Quesnel AM Ishai R McKenna MJ. Otosclerosis: temporal bone pathology. Otolaryngol Clin N Am. (2018) 51:291–303. doi: 10.1016/j.otc.2017.11.001, PMID: 29397947

[12] Atan D İkincioğulları A Özcan KM Köseoğlu S. New predictive parameters of bell’s palsy: neutrophil to lymphocyte ratio and platelet to lymphocyte ratio. Balkan Med J. (2015) 32:167–70. doi: 10.5152/balkanmedj.2015.15456, PMID: 26167340 PMC4432696

[13] Damar M Dinç AE Erdem D Aydil U Kizil Y Eravcı FC. Pretreatment neutrophil-lymphocyte ratio in salivary gland tumors is associated with malignancy. Otolaryngol Head Neck Surg. (2016) 155:988–96. doi: 10.1177/0194599816659257, PMID: 27436419

[14] Atan D Apaydın E Özcan KM Dere H. New diagnostic indicators in chronic otitis media with effusion: neutrophil to lymphocyte ratio and thrombocyte lymphocyte ratio. ENT Updates. (2016) 6:12–5. doi: 10.2399/jmu.2016001007, PMID: 23711010

[15] Boztepe OF Demir M Gün T Bilal N Ensari NA Doğru H. A novel predictive marker for the viscosity of otitis media with effusion. Int J Pediatr Otorhinolaryngol. (2015) 79:2355–8. doi: 10.1016/j.ijporl.2015.10.043, PMID: 26590001

[16] Kara A Guven M Yilmaz MS Demir D Elden H. Are neutrophil, platelet and eosinophil-to-lymphocyte ratio and red blood cell distribution width can be used for nasal polyposis? Eur Arch Otorrinolaringol. (2018) 275:409–13. doi: 10.1007/s00405-017-4821-3, PMID: 29192331

[17] Kuzucu İ Güler İ Kum RO Baklacı D Özcan M. Increased neutrophil lymphocyte ratio and platelet lymphocyte ratio in malignant parotid tumors. Braz J Otorhinolaryngol. (2020) 86:105–10. doi: 10.1016/j.bjorl.2019.02.009, PMID: 31122885 PMC9422377

[18] Kum RO Ozcan M Baklaci D Yurtsever Kum N Yilmaz YF Unal A. Investigation of neutrophil-to-lymphocyte ratio and mean platelet volume in sudden hearing loss. Braz J Otorhinolaryngol. (2015) 81:636–41. doi: 10.1016/j.bjorl.2015.08.009, PMID: 26480902 PMC9442714

[19] Saeki K Miura Y Aki D Kurosaki T Yoshimura A. The B cell specific major raft protein, raftlin, is necessary for the integrity of lipid raft and BCR signal transduction. EMBO J. (2003) 22:3015–26. doi: 10.1093/emboj/cdg293, PMID: 12805216 PMC162145

[20] Lee W Yoo H Ku SK Kim SW Bae JS. Raftlin: a new biomarker in human sepsis. Inflammation. (2014) 37:706–11. doi: 10.1007/s10753-013-9788-7, PMID: 24317805

[21] Tatematsu M Yoshida R Morioka Y Ishii N Funami K Watanabe A. Raftlin controls lipopolysaccharide-induced TLR4 internalization and TICAM-1 signaling in a cell type-specific manner. J Immunol. (2016) 196:3865–76. doi: 10.4049/jimmunol.1501734, PMID: 27022195

[22] Ozer A Tolun F Aslan F Hatırnaz S Alkan F. The role of G protein-associated estrogen receptor (GPER) 1, corin, raftlin, and estrogen in etiopathogenesis of intrauterine growth retardation. J Matern Fetal Neonatal Med. (2019) 34:755–60. doi: 10.1080/14767058.2019.1615433 31088311

[23] Bilgen F Ural A Kurutas EB Bekerecioglu M. The effect of oxidative stress and Raftlin levels on wound healing. Int Wound J. (2019) 16:1178–84. doi: 10.1111/iwj.13177, PMID: 31407472 PMC7948575

[24] Ulu S Ulu MS Bucak A Ahsen A Yucedag F Aycicek A. Neutrophil-to-lymphocyte ratio as a new, quick, and reliable indicator for predicting diagnosis and prognosis of idiopathic sudden sensorineural hearing loss. Otol Neurotol. (2013) 34:1400–4. doi: 10.1097/MAO.0b013e31829b57df, PMID: 23988996

[25] Yazici S Yazici M Erer B Calik Y Ozhan H Ataoglu S. The platelet indices in patients with rheumatoid arthritis: mean platelet volume reflects disease activity. Platelets. (2010) 21:122–5. doi: 10.3109/09537100903474373, PMID: 20050760

[26] Somuk BT Soyalıç H Koc S Gürbüzler L Doğru S Eyibilen A. Mean platelet volume as an inflammatory marker of chronic otitis media with effusion. Int J Pediatr Otorhinolaryngol. (2014) 78:1958–60. doi: 10.1016/j.ijporl.2014.08.037, PMID: 25200601

[27] Lin YT Tsai MH Su YY Chen WC Huang SC Chien CY. Expression of major lipid raft protein Raftlin in chronic rhinosinusitis with nasal polyps in smoking and non-smoking patients correlated with Interleukin-17 and tumor necrosis factor-α levels. Biomolecules. (2022) 12:1316. doi: 10.3390/biom12091316, PMID: 36139155 PMC9496107

[28] Hursitoglu O Kurutas EB Strawbridge R Oner E Gungor M Tuman TC. Serum NOX1 and Raftlin as new potential biomarkers of major depressive disorder: a study in treatment-naive first episode patients. Prog Neuro-Psychopharmacol Biol Psychiatry. (2023) 121:110670. doi: 10.1016/j.pnpbp.2022.110670, PMID: 36341844

[29] Hurşitoğlu O Kurutas EB Strawbridge R Uygur OF Yildiz E Reilly TJ. Serum NOX1 and Raftlin as new potential biomarkers of interest in schizophrenia: a preliminary study. Neuropsychiatr Dis Treat. (2022) 18:2519–27. doi: 10.2147/NDT.S385631, PMID: 36349345 PMC9637347

[30] Demirhan İ Oner E Yuksel Z Yuksel M Belge Kurutas E. Raftlin and 8-iso-prostaglandin F2α levels and gene network analysis in patients with Modic changes. Eur Spine J. (2023) 32:2368–76. doi: 10.1007/s00586-023-07757-7, PMID: 37208489

[31] Lin F Yan L Yuan X Yang X Yang X Yang Y. Implications of Raftlin in different diseases: from molecular biology to diagnostic value. Biomark Med. (2025) 19:91–9. doi: 10.1080/17520363.2025.2453411, PMID: 39840913 PMC11792867

[32] Saeki K Fukuyama S Ayada T Nakaya M Aki D Takaesu G. A major lipid raft protein Raftlin modulates T cell receptor signaling and enhances Th17-mediated autoimmune responses. J Immunol. (2009) 182:5929–37. doi: 10.4049/jimmunol.0802672 19414744

